# Refinements and Generalizations of the Shannon Lower Bound via Extensions of the Kraft Inequality

**DOI:** 10.3390/e28010076

**Published:** 2026-01-09

**Authors:** Neri Merhav

**Affiliations:** The Viterbi Faculty of Electrical and Computer Engineering, Technion—Israel Institute of Technology, Technion City, Haifa 3200003, Israel; merhav@ee.technion.ac.il

**Keywords:** rate-distortion theory, Shannon lower bound, Kraft’s inequality, coding redundancy, Lempel-Ziv algorithm

## Abstract

We derive a few extended versions of the Kraft inequality for lossy compression, which pave the way to the derivation of several refinements and extensions of the well-known Shannon lower bound in a variety of instances of rate-distortion coding. These refinements and extensions include sharper bounds for one-to-one codes and *D*-semifaithful codes, a Shannon lower bound for distortion measures based on sliding-window functions, and an individual-sequence counterpart of the Shannon lower bound.

## 1. Introduction

The Shannon lower bound (SLB) is one of the most important analytic tools in rate-distortion theory because it provides a simple, explicit, and often very tight lower bound to the rate-distortion function for a wide class of sources and distortion measures; see, e.g., Subsection 3.4.1 of [1], Sections 4.3 and 4.6 of [2], and Problem 10.6 of [3]. Its significance lies in giving a universal benchmark that connects rate-distortion tradeoffs to the entropy or the differential entropy of the source (depending on whether the source has a discrete or continuous alphabet), thereby offering an intuitively transparent approximation in regimes where exact evaluation of the rate-distortion function is intractable. The SLB is particularly powerful at low distortion, where it frequently coincides with the exact rate-distortion function for smooth sources, and it serves as a foundation for many refinements and asymptotic approximations (e.g., high-resolution analysis, corrections for lattice quantizers, and recent non-asymptotic bounds). In fact, the literature contains reported results on the asymptotic tightness of the SLB in the limit of low distortion under fairly mild regularity conditions [4,5]. Because computing the rate-distortion function exactly is generally difficult, the SLB plays a central role in the analysis, design, and performance assessment of lossy compression schemes. More recent studies include the finite block-length regime [6,7] and further developments concerning the quadratic distortion function [8].

In [9] Campbell derived an extension of Kraft’s inequality that leads to the SLB in both the discrete and the continuous alphabet cases. However, his results were claimed to apply to rate-distortion codes whose distortion level is defined by the distance between the two most distant source-space vectors that are mapped to the same codeword, namely, the diameter (as opposed to the radius) of the distortion ball centered at the reproduction vector. In [10], a similar Kraft inequality was derived for *D*-semifaithful codes, namely, codes that incur per-letter distortion that never exceeds *D* in the usual sense. An additional benefit of the derivation in [10] was that it could also deliver an $O\left(\frac{\log n}{n}\right)$ redundancy term on top of the SLB, at least for certain distortion measures for which there is an explicit expression of the cardinality (or the volume, in the continuous case) of a ball of normalized radius *D* in the source vector space.

In this work, we propose a few other extended versions of Kraft’s inequality that together pave the way to several further refinements and generalizations of the SLB. In these extensions of Kraft’s inequality, the idea is to upper bound the summation (or the integral, in the continuous case) of an exponentiated negative linear combination of the code length and the distortion incurred by each and every vector in the source space. By contrasting an upper bound with a lower bound to this quantity, we obtain several refinements and extensions to the SLB, which apply to the following scenarios.

1. *One-to-one codes.* Instead of assuming that the reproduction vectors are represented by uniquely decodable (UD) codes, we relax this restriction and allow any one-to-one code in the level of *n*-vectors. The lower bound then becomes the SLB minus an $O\left(\frac{\log n}{n}\right)$ term, similarly to the lossless case derived by Rissanen [11].

2. *D-semifaithful codes.* Similarly to [10], we consider *D*-semifaithful codes, but here we allow several simultaneous distortion criteria. Using saddle-point integration, we show that the resulting lower bound is given by the SLB plus $\frac{k\log n}{2n}+o\left(\frac{1}{n}\right)$, where *k* is the “effective number” of distortion constraints. The concrete meaning of this term will be clarified in Section 4.2 (see the paragraph that includes Equation (40)). Generally speaking, it means the remaining number of independent constraints after removing the redundant (inactive) ones. This $O\left(\frac{\log n}{n}\right)$ redundancy term stems from a more accurate evaluation of the volume of a ball of radius nD around each source word, compared to the one obtained by Chernoff bounding techniques and by the method of types in the finite-alphabet case. Interestingly, the $O\left(\frac{\log n}{n}\right)$ behavior is also coherent with that of earlier derived lower bounds to the rate-distortion function for *n*-blocks in the finite-alphabet case; see, e.g., [12] and references therein.

3. *Sliding-window distortion functions.* In some applications, one might wish to shape the spectrum or the memory properties of the reconstruction error signal. This can be performed by imposing additional distortion constraints defined by additive functions that operate on two or more consecutive samples of the error signal in a sliding-window fashion. Our framework is capable of incorporating such distortion functions and allowing a derivation of the generalized SLB for this case.

4. *Individual sequences and finite-state encoders.* By developing a generalized Kraft inequality, similar (but not identical) to the one by Ziv and Lempel [13] for finite-state encoders, we derive also an individual-sequence counterpart of the SLB, where the source entropy term is replaced by the Lempel–Ziv complexity.

As is well known, the classical SLB can be obtained significantly more simply and easily than going via the Kraft inequality. In particular, it is obtained by a straightforward direct manipulation of the mutual information. But it should be emphasized that the point of this article is not a quest for a simpler proof of the SLB. The point is that the path that goes via the Kraft inequality leads to the above-mentioned extensions and refinements.

The outline of the remaining part of this article is as follows. In Section 2, we establish some notation conventions (Section 2.1) and provide elementary background on the SLB (Section 2.2). In Section 3, we present and prove our extended Kraft inequality in several variations. In Section 4, we derive corresponding lower bounds, first, for UD lossless compression of the reproduction data, then for one-to-one compression thereof (Section 4.1), and finally, for *D*-semifaithful codes (Section 4.2). In Section 5, we address the case of sliding-window distortion functions. In Section 6, we first provide some background on finite-state compression of individual sequences and the LZ algorithm (Section 6.1) and then derive an individual-sequence counterpart of the SLB (Section 6.2). Finally, in Section 6.2, we summarize and conclude this paper.

## 2. Notation Conventions and Background

### 2.1. Notation Conventions

Throughout this paper, scalar random variables (RVs) will be denoted by capital letters, their sample values will be denoted by the respective lower-case letters, and their alphabets will be denoted by the respective calligraphic letters. A similar convention will apply to random vectors and their sample values, which will be denoted with same symbols superscripted by the dimension. Thus, for example, $U^{n}$ (*n*—positive integer) will denote a random *n*-vector $\left(U_{1},\ldots ,U_{n}\right)$, and $u^{n}=\left(u_{1},\ldots ,u_{n}\right)$ is a specific vector value in $U^{n}$, the *n*–th Cartesian power of U, which is the alphabet of each component of $u^{n}$. In some of our derivations below, there will be a need to refer to multiple copies of the vector $u^{n}$. In such cases, in order to avoid cumbersome subscripts and superscripts for indexing, we will use the alternative notation *u* for $u^{n}$, and then the various copies will be denoted by $u_{1}$, $u_{2}$, etc. Returning to the first notation method, $u_{i}^{j}$ and $U_{i}^{j}$, where *i* and *j* are integers and i≤j, will designate segments $\left(u_{i},\ldots ,u_{j}\right)$ and $\left(U_{i},\ldots ,U_{j}\right)$, respectively, where for i=1, the subscript will be omitted (as above). For i>j, $u_{i}^{j}$ (or $U_{i}^{j}$) will be understood as the null string. Logarithms and exponents, throughout this paper, will be understood to be taken to the base 2 unless specified otherwise. The indicator function of an event A will be denoted by I{A}, i.e., I{A}=1 if A occurs and I{A}=0 if not.

Sources and probability distributions associated with them will be denoted generically by the letter *P* subscripted by the name of the RV and its conditioning, if applicable, exactly like in ordinary textbook notation standards; e.g., $P_{U^{n}}\left(u^{n}\right)$ is the probability function of $U^{n}$ at the point $U^{n}=u^{n}$, $P_{X|U^{n}}\left(x|u^{n}\right)$ is the conditional probability of X=x given $U^{n}=u^{n}$, and so on. Whenever clear from the context, these subscripts will be omitted. Information theoretic quantities, like entropies and mutual informations, will be denoted following the usual conventions of the information theory literature, e.g., $H(U^{n})$, $I(S;U^{n}|V^{n})$, and so on. The differential entropy of a continuous valued RV, $U^{n}$, will be denoted by $h(U^{n})$. The expectation operator will be denoted by E{·} and the probability of an event A will be denoted by Pr{A}.

It should be noted that our derivations will apply to both discrete-alphabet sources and to continuous-alphabet sources. To avoid repetitions, we henceforth carry on under the assumption of a continuous-alphabet source with the understanding that in the discrete-alphabet cases, integrations over $U^{n}$ should simply be replaced by summations.

Let $U^{n}=\left(U_{1},U_{2},\ldots ,U_{n}\right)$ denote a source vector, drawn from a stochastic process, *P*, whose alphabet is U. The source vector is compressed by a lossy fixed-to-variable (F-V) source code, defined by an encoder $\phi_{n}:U^{n}\to B_{n}\subset {\left\{0,1\right\}}^{★}$ and a decoder $\psi_{n}:B_{n}\to V_{n}\subseteq U^{n}$, where $B_{n}$ denotes a certain subset of the set of all binary variable-length strings, {0,1}*. Without loss of generality (and optimality), it is assumed that the encoder $\phi_{n}$ can be viewed as a cascade of a reproduction encoder (vector quantizer) $U^{n}\to V_{n}$ and a uniquely decodable (UD) lossless code $V_{n}\to B_{n}$. In a certain part of our results, this unique decodability assumption will be partially relaxed to become the less demanding assumption of a one-to-one mapping. Let $L[\phi_{n}\left(u^{n}\right)]$ denote the length (in bits) of the compressed codeword, $\phi_{n}\left(u^{n}\right)$.

Assuming that U is a group with certain addition and subtraction operations (e.g., modulo-*K* addition/subtraction for U={0,1,…,K−1} for a finite positive integer *K* or ordinary addition/subtraction for U=R) we will focus on additive difference distortion measures, where the distortion between the source vector $u^{n}\in U^{n}$ and the reproduction vector $v^{n}=\psi_{n}\left(\phi_{n}\left(u^{n}\right)\right)\in V_{n}$ will be given by

(1) $$d\left(u^{n},v^{n}\right)=\sum_{i=1}^{n}d\left(u_{i},v_{i}\right)=\sum_{i=1}^{n}\rho \left(u_{i}-v_{i}\right),$$

where ρ(z), z∈U is a certain non-negative function, which vanishes if and only if z=0, for example, ρ(z)=|z|, $\rho \left(z\right)=z^{2}$, etc. For the sake of convenience, we will sometimes denote $d(u^{n},v^{n})$ by $\rho (u^{n}-v^{n})$, which for a given encoder–decoder pair is also $\rho (u^{n}-\psi_{n}\left(\phi_{n}\left(u^{n}\right)\right))$. Given an encoder–decoder pair, $\left(\phi_{n},\psi_{n}\right)$, the expected distortion, $\sum_{i=1}^{n}E\left\{\rho \left(U_{i}-V_{i}\right)\right\}$, will be constrained to be less than or equal to nD, where D>0 designates the per-letter distortion level allowed. In certain parts of our derivations, a more restrictive pointwise distortion constraint will be imposed, i.e., $\rho (u^{n}-\psi_{n}\left(\phi_{n}\left(u^{n}\right)\right))\leq nD$ for all $u^{n}\in U^{n}$. In other parts, more than one difference distortion measure will play a role, and accordingly, more than one distortion constraint will be imposed. Given *k* difference distortion functions, $\rho_{j}\left(\cdot \right)$, j=1,2…,k, we then require $E\left\{\rho_{j}\left(U^{n}-\psi_{n}\left(\phi_{n}\left(U^{n}\right)\right)\right)\right\}\leq nD_{j}$ (or $\max_{u^{n}\in U^{n}}\rho_{j}\left(u^{n}-\psi_{n}\left(\phi_{n}\left(u^{n}\right)\right)\right)\}\leq nD_{j}$) for all j=1,2,…,k. In another part of our results, we also allow distortion functions to be additive sliding-window functions operating on *m* consecutive symbols of the difference $z^{n}=u^{n}-v^{n}$. I.e.,

(2) $$\rho \left(z^{n}\right)=\sum_{i=m}^{n}\rho \left(z_{i-m+1}^{i}\right).$$

This corresponds to situations where we wish to shape not only the ‘intensity’ of the error signal, $z^{n}$, but also its memory properties, for example, the correlations between consecutive symbols of $z^{n}$. We will elaborate more on this in Section 5.

Similarly to $u^{n}$, for which we adopt the alternative notation *u*, the same will apply to $v^{n}$, which will also be denoted by *v*, along with its multiple copies $v_{1}$, $v_{2}$, and so on.

### 2.2. Background

For a continuous-alphabet memoryless source *P*, the SLB is given by

(3) $$R\left(D\right)\geq R_{SLB}\left(D\right)\overset{▵}{=}h\left(U\right)-\Phi \left(D\right),$$

where h(U) is the differential entropy of a single symbol *U* and

(4) $$\Phi \left(D\right)\overset{▵}{=}\underset{\left\{Z:\;E\{\rho (Z)\}\leq D\right\}}{\sup}h\left(Z\right)=\underset{\beta \geq 0}{\inf}\left\{\beta D+\log\left[\int_{U}2^{-\beta \rho (z)}dz\right]\right\},$$

assuming that $\int_{U}2^{-\beta \rho (z)}dz<\infty$ for some β>0. The equivalence between the two expressions of Φ(D) can be easily shown using standard techniques. For the sake of completeness, we prove this equivalence in Appendix A (see also Section 4.3.1 in [1] and Sections 4.3 and 4.6 in [2]). The advantage of the second formula of Φ(D) is that it involves optimization over one parameter only, as opposed to variational calculus in the first formula. Clearly, if the source is discrete rather than continuous, the differential entropy, h(U), should be replaced by ordinary entropy, H(U), and the integration over U should be replaced by summation, as indicated above.

For a source with memory, the SLB is given by

(5) $$R_{n}\left(D\right)\overset{▵}{=}\min_{\{P_{V^{n}|U^{n}}:\;E\left\{\rho \left(U^{n}-V^{n}\right)\leq nD\right\}}\frac{I(U^{n};V^{n})}{n}\geq \frac{h(U^{n})}{n}-\Phi \left(D\right),$$

which in the limit of large *n* becomes

(6) $$R\left(D\right)\geq \lim_{n\to \infty }\frac{h(U^{n})}{n}-\Phi \left(D\right)\overset{▵}{=}\bar{h}\left(U^{\infty }\right)-\Phi \left(D\right),$$

where $\bar{h}\left(U^{\infty }\right)$ is the differential entropy rate of the source. In all cases, the function Φ(D) remains as in (4).

## 3. Extended Kraft Inequalities

For a given encoder–decoder pair, $\left(\phi_{n},\psi_{n}\right)$, and parameters α>1 and β≥0, define the *extended Kraft integral* (or the extended Kraft sum, in the discrete-alphabet case) as follows:

(7) $$Z^{n}\left(\alpha ,\beta \right)\overset{▵}{=}\int_{U^{n}}\exp_{2}\left\{-\alpha L\left[\phi_{n}\left(u^{n}\right)\right]-\beta \rho \left(u^{n}-\psi_{n}\left(\phi_{n}\left(u^{n}\right)\right)\right)\right\}du^{n}.$$

**Lemma** **1.** *Let $\left(\phi_{n},\psi_{n}\right)$ induce a UD lossless compression of $v^{n}=\psi_{n}\left(\phi_{n}\left(u^{n}\right)\right)$. Then, for every α>1 and β≥0,*

(8) $$Z^{n}\left(\alpha ,\beta \right)\leq {\left[\int_{U}2^{-\beta \rho (z)}dz\right]}^{n}.$$

**Proof of Lemma** **1.** Let us examine the expression of ${\left[Z^{n}\left(\alpha ,\beta \right)\right]}^{k}$ for an arbitrary positive integer *k*.

(9) $$\begin{array}{ccc} {\left[Z^{n}\left(\alpha ,\beta \right)\right]}^{k} & = & {\left[\int_{U^{n}}\exp_{2}\left\{-\alpha L\left[\phi_{n}\left(u\right)\right]-\beta \rho \left(u-\psi_{n}\left(\phi_{n}\left(u\right)\right)\right)\right\}du\right]}^{k}\\ & = & \int_{U^{n}}du_{1}\cdot \cdot \cdot \int_{U^{n}}du_{k}\cdot \exp_{2}\left\{-\alpha \sum_{i=1}^{k}L\left[\phi_{n}\left(u_{i}\right)\right]-\beta \sum_{i=1}^{k}\rho \left(u_{i}-\psi_{n}\left(\phi_{n}\left(u_{i}\right)\right)\right)\right\}\\ & \leq & \sum_{l=1}^{\infty }\sum_{\left\{{\left\{v_{i}\right\}}_{i=1}^{k}:\;\sum_{i=1}^{k}L\left(v_{i}\right)]=l\right\}}\int_{\left\{u_{1}:\;\psi_{n}\left(\phi_{n}\left(u_{1}\right)\right)=v_{1}\right\}}du_{1}\cdot \cdot \cdot \int_{\left\{u_{k}:\;\psi_{n}\left(\phi_{n}\left(u_{k}\right)\right)=v_{k}\right\}}du_{k}\times \\ & & \exp_{2}\left\{-\alpha l-\beta \sum_{i=1}^{k}\rho \left(u_{i}-v_{i}\right)\right\}\\ & \leq & \sum_{l=1}^{\infty }2^{-\alpha l}\sum_{\left\{{\left\{v_{i}\right\}}_{i=1}^{k}:\;\sum_{i=1}^{k}L\left(v_{i}\right)=l\right\}}\int_{U^{n}}dz_{1}\ldots \int_{U^{n}}dz_{k}\exp_{2}\left\{-\beta \sum_{i=1}^{k}\rho \left(z_{i}\right)\right\}\\ & \leq & \sum_{l=1}^{\infty }2^{-\alpha l}\cdot 2^{l}{\left[\int_{U^{n}}dz^{n}\exp_{2}\left\{-\beta \rho \left(z^{n}\right)\right\}\right]}^{k}\\ & = & {\left[\int_{U^{n}}\cdot dz^{n}\exp_{2}\left\{-\beta \rho \left(z^{n}\right)\right\}\right]}^{k}\cdot \sum_{l=1}^{\infty }2^{-(\alpha -1)l}\\ & = & \frac{{\left[\int_{U}dz\exp_{2}\left\{-\beta \rho \left(z\right)\right\}\right]}^{nk}}{2^{\alpha -1}-1}, \end{array}$$

where in the second inequality we transformed each integration variable $u_{i}$ into $z_{i}=u_{i}-v_{i}$ (a transformation with unit Jacobian) and expanded the integration domain from $\left\{u_{i}:\psi_{n}\left(\phi_{n}\left(u_{i}\right)\right)=v_{i}\right\}$ to $U^{n}$. Consequently,

(10) $$Z^{n}\left(\alpha ,\beta \right)\leq \frac{{\left[\int_{U}dz\exp_{2}\left\{-\beta \rho \left(z\right)\right\}\right]}^{n}}{{\left(2^{\alpha -1}-1\right)}^{1/k}},$$

which upon taking the limit k→∞ becomes

(11) $$Z^{n}\left(\alpha ,\beta \right)\leq {\left[\int_{U}2^{-\beta \rho (z)}dz\right]}^{n},$$

completing the proof of Lemma 1. □

For one-to-one codes, instead of taking the limit of k→∞ in the proof of Lemma 1, we simply set k=1, since the one-to-one property is merely imposed at the level of a single *n*-block rather than at the level of concatenations of blocks, as in UD codes. This yields the following variation of Lemma 1.

**Lemma** **2.** *Let $\left(\phi_{n},\psi_{n}\right)$ induce a one-to-one mapping between $\psi_{n}\left(u^{n}\right)$ and $v^{n}=\psi_{n}\left(\phi_{n}\left(u^{n}\right)\right)$. Then, for every α>1 and β≥0,*

(12) $$Z_{1-1}^{n}\left(\alpha ,\beta \right)\leq \frac{{\left[\int_{U}2^{-\beta \rho (z)}dz\right]}^{n}}{2^{\alpha -1}-1},$$

*where* $Z_{1-1}^{n}\left(\alpha ,\beta \right)$ *is defined exactly as* $Z^{n}\left(\alpha ,\beta \right)$*, except that it may apply to the larger class of one-to-one codes, rather than UD codes.*

When α∈(1,2), the denominator, $2^{\alpha -1}-1$, is smaller than unity, and then the upper bound to $Z_{1-1}^{n}\left(\alpha ,\beta \right)$ in Lemma 2 is larger than that of Lemma 1. Indeed, the interesting region for selecting the values of α is in the vicinity of unity, where $2^{\alpha -1}-1<1$.

Returning to the class of UD lossless encodings of $v^{n}=\psi_{n}\left(\phi_{n}\left(u^{n}\right)\right)$, two additional variations of the above extended Kraft inequality can be considered. The first pertains to *D*-semifaithful codes, namely, codes for which the distortion is restricted to never exceed nD, pointwise, and not merely in expectation, that is, $\max_{u^{n}\in U^{n}}\rho \left(u^{n}-\psi_{n}\left(\phi_{n}\left(u^{n}\right)\right)\right)\leq nD$, where *D* is the allowed per-letter distortion. The second variation is associated with fixed-rate codes, i.e., codes for which $L[\phi_{n}\left(u^{n}\right)]=nR$ for all $u^{n}\in U^{n}$, where R>0 is the allowed coding rate.

For *D*-semifaithful codes, let us redefine the integrand of the extended Kraft integral by replacing the term $\beta \rho (u^{n}-\psi_{n}\left(\phi_{n}\left(u^{n}\right)\right))$ in the exponent with the function $W(\rho \left(u^{n}-\psi_{n}\left(\phi_{n}\left(u^{n}\right)\right)\right)-nD)$, where W[·] is the infinite well function (IWF),

(13) $$W\left(t\right)\overset{▵}{=}\left\{\begin{array}{cc} 0 & t\leq 0\\ \infty & t>0 \end{array}\right.$$

This causes the integrand of the extended Kraft integral to vanish wherever the distortion constraint is violated, and then the extended Kraft integral becomes

(14) $$Z_{D-sf}^{n}\left(\alpha \right)\overset{▵}{=}\int_{S_{n}\left(D\right)}\exp_{2}\left\{-\alpha L\left[\phi_{n}\left(u^{n}\right)\right]\right\}du^{n},$$

where $S_{n}\left(D\right)=\left\{u^{n}:\;\rho \left(u^{n}-\psi_{n}\left(\phi_{n}\left(u^{n}\right)\right)\right)\leq nD\right\}$. In this case, a simple modification of the proof of Lemma 1 yields the following version of the extended Kraft inequality (see also [10]).

**Lemma** **3.** *Let $\left(\phi_{n},\psi_{n}\right)$ be a D-semifaithful code that comprises UD lossless encoding of $v^{n}=\psi_{n}\left(\phi_{n}\left(u^{n}\right)\right)$. Then, for every α>1,*

(15) $$Z_{D-sf}^{n}\left(\alpha \right)\leq Vol\left\{z^{n}:\;\rho \left(z^{n}\right)\leq nD\right\}.$$

Here too, if the UD property is replaced by the one-to-one property, then the right-hand side should be divided by $2^{\alpha -1}-1$.

Finally, for fixed-rate codes, let us replace the term $L[\phi_{n}\left(u^{n}\right)]$ of the Kraft integrand by nR and return the second term therein to $\beta \rho (u^{n}-\psi_{n}\left(\phi_{n}\left(u^{n}\right)\right))$. By similar manipulations of the proof of Lemma 1, we find that for fixed-rate codes, the Kraft integral becomes

(16) $$Z_{\mathrm{fr}}^{n}\left(\alpha ,\beta \right)\overset{▵}{=}\int_{U^{n}}\exp_{2}\left\{-\alpha nR-\beta \rho \left(u^{n}-\psi_{n}\left(\phi_{n}\left(u^{n}\right)\right)\right)\right\}du^{n},$$

with the following version of the extended Kraft inequality.

**Lemma** **4.** *Let $\left(\phi_{n},\psi_{n}\right)$ be a rate-R fixed-rate code. Then, for every α≥0 and β≥0,*

(17) $$Z_{fr}^{n}\left(\alpha ,\beta \right)\leq 2^{n(1-\alpha )R}\cdot {\left[\int_{U}2^{-\beta \rho (z)}dz\right]}^{n}.$$

## 4. Lower Bounds

To fix ideas, we first demonstrate how the classical SLB is obtained from Lemma 1. Consider an arbitrary encoder–decoder pair, $\left(\phi_{n},\psi_{n}\right)$, in which the reproduction vector, $v^{n}=\psi_{n}\left(\phi_{n}\left(u^{n}\right)\right)$, is losslessly compressed by a UD code. Then, following Lemma 1, we have the following chain of inequalities:

(18) $$\begin{array}{ccc} {\left[\int_{U}2^{-\beta \rho (z)}dz\right]}^{n} & \overset{\left(a\right)}{\geq } & Z_{n}\left(\alpha ,\beta \right)\\ & = & \int_{U^{n}}\exp_{2}\left\{-\alpha L\left[\phi_{n}\left(u^{n}\right)\right]-\beta \rho \left(u^{n}-\psi_{n}\left(\phi_{n}\left(u^{n}\right)\right)\right)\right\}du^{n}\\ & = & \int_{U^{n}}P\left(u^{n}\right)\exp_{2}\left\{-\alpha L\left[\phi_{n}\left(u^{n}\right)\right]-\beta \rho \left(u^{n}-\psi_{n}\left(\phi_{n}\left(u^{n}\right)\right)\right)-\log P\left(u^{n}\right)\right\}du^{n}\\ & = & E\left\{\exp_{2}\left\{-\alpha L\left[\phi_{n}\left(U^{n}\right)\right]-\beta \rho \left(U^{n}-\psi_{n}\left(\phi_{n}\left(U^{n}\right)\right)\right)-\log P\left(U^{n}\right)\right\}\right\}\\ & \overset{\left(b\right)}{\geq } & \exp_{2}\left[-\alpha E\left\{L\left[\phi_{n}\left(U^{n}\right)\right]\right\}-\beta E\left\{\rho \left(U^{n}-\psi_{n}\left(\phi_{n}\left(U^{n}\right)\right)\right)\right\}-E\left\{\log P\left(U^{n}\right)\right\}\right]\\ & = & \exp_{2}\left[-\alpha E\left\{L\left[\phi_{n}\left(U^{n}\right)\right]\right\}-\beta E\left\{\rho \left(U^{n}-\psi_{n}\left(\phi_{n}\left(U^{n}\right)\right)\right)\right\}+h\left(U^{n}\right)\right], \end{array}$$

where (a) stems from Lemma 1 and (b) is due to Jensen’s inequality applied to the convex function, $f\left(t\right)=2^{t}$. It follows that

(19) $$\alpha E\left\{L\left[\phi_{n}\left(U^{n}\right)\right]\right\}+\beta E\left\{\rho \left(U^{n}-\psi_{n}\left(\phi_{n}\left(U^{n}\right)\right)\right)\right\}\geq h\left(U^{n}\right)-n\log\left[\int_{U}2^{-\beta \rho (z)}dz\right].$$

Since this holds true for every α>1 while the right-hand side is independent of α, we may take the infimum of the left-hand side in the range α>1 to obtain, after normalization by *n*,

(20) $$\frac{E\{L\left[\phi_{n}\left(U^{n}\right)\right]\}}{n}+\beta \cdot \frac{E\{\rho \left(U^{n}-\psi_{n}\left(\phi_{n}\left(U^{n}\right)\right)\right)\}}{n}\geq \frac{h(U^{n})}{n}-\log\left[\int_{U}2^{-\beta \rho (z)}dz\right].$$

At this point, there are several possible perspectives that can be adopted regarding Equation (20). The first is, of course, to view this as a lower bound to the Lagrangian of rate and distortion. The second is to impose an expected distortion constraint, $E\{\rho \left(U^{n}-\psi_{n}\left(\phi_{n}\left(U^{n}\right)\right)\right)\}\leq nD$, and then Equation (20) would yield a lower bound to the expected rate according to

(21) $$\frac{E\{L\left[\phi_{n}\left(U^{n}\right)\right]\}}{n}\geq \frac{h(U^{n})}{n}-\log\left[\int_{U}2^{-\beta \rho (z)}dz\right]-\beta D.$$

Since this holds true for every β≥0 while the left-hand side is independent of β, we may maximize the right-hand side over β≥0, to obtain

(22) $$\frac{E\{L\left[\phi_{n}\left(U^{n}\right)\right]\}}{n}\geq \frac{h(U^{n})}{n}-\underset{\beta \geq 0}{\inf}\left\{\log\left[\int_{U}2^{-\beta \rho (z)}dz\right]+\beta D\right\}=\frac{h(U^{n})}{n}-\Phi \left(D\right),$$

thus recovering the classical SLB. In case of multiple distortion constraints, say,

(23) $$E\left\{\rho_{j}\left(U^{n}-\psi_{n}\left(\phi_{n}\left(U^{n}\right)\right)\right)\right\}\leq nD_{j},\;\;\;\;\;j=1,2,\ldots ,k,$$

the above rate lower bound continues to apply, provided that β, *D* and ρ(z) are redefined as *k*-dimensional vectors, $\beta =(\beta_{1},\ldots ,\beta_{k})$, $D=(D_{1},\ldots ,D_{k})$, and $\rho \left(z\right)=(\rho_{1}\left(z\right),\ldots ,\rho_{k}\left(z\right))$, and accordingly, βD and βρ(z) are understood to be inner products. The infimum over β is then defined across ${\left[0,\infty \right)}^{k}$.

Returning to Equation (20) and to a single distortion criterion, we may alternatively apply a rate constraint $E\{L\left[\phi_{n}\left(U^{n}\right)\right]\}\leq nR$ and then obtain a lower bound to the expected distortion:

(24) $$\begin{array}{ccc} \frac{E\{\rho \left(U^{n}-\psi_{n}\left(\phi_{n}\left(U^{n}\right)\right)\right)\}}{n} & \geq & \underset{\beta \geq 0}{\sup}\frac{1}{\beta }\left(\frac{h(U^{n})}{n}-\log\left[\int_{U}2^{-\beta \rho (z)}dz\right]-R\right)\\ & = & \underset{\gamma \geq 0}{\sup}\gamma \left(\frac{h(U^{n})}{n}-\log\left[\int_{U}2^{-\rho (z)/\gamma }dz\right]-R\right), \end{array}$$

which is the distortion-rate counterpart of the SLB.

Having established the classical SLB for general variable-rate codes with UD lossless compression of the reproduction vectors, we now carry on to derive some refinements and extensions associated with the other types of codes that we mentioned. The idea would be to apply the same chain of inequalities as in (18) but to invoke Lemma 2 or Lemma 3, or Lemma 4, according to the relevant class of codes, instead of Lemma 1. We next implement this plan for one-to-one codes and for *D*-semifaithful codes. The same methodology can be applied to fixed-rate codes, but we will not delve into this here.

### 4.1. One-to-One Codes

For one-to-one codes, we repeat the same derivation as in (18) by invoking Lemma 2 instead of Lemma 1. This yields the following modified version of Equation (19):

(25) $$\alpha E\left\{L\left[\phi_{n}\left(U^{n}\right)\right]\right\}+\beta E\left\{\rho \left(U^{n}-\psi_{n}\left(\phi_{n}\left(U^{n}\right)\right)\right)\right\}\geq h\left(U^{n}\right)-n\log\left[\int_{U}2^{-\beta \rho (z)}dz\right]+\log\left(2^{\alpha -1}-1\right).$$

Applying the average distortion constraint, optimizing over β, and normalizing by *n*, we obtain

(26) $$\frac{E\{L\left[\phi_{n}\left(U^{n}\right)\right]\}}{n}\geq \underset{\alpha \geq 1}{\sup}\left\{\frac{h\left(U^{n}\right)/n-\Phi \left(D\right)}{\alpha }+\frac{\log(2^{\alpha -1}-1)}{\alpha n}\right\}.$$

The maximization of the right-hand side with respect to α does not seem to lend itself to a closed-form expression, but by selecting $\alpha =\alpha_{n}=1+c\frac{\log n}{n}$ (*c* being an arbitrary positive constant), it is readily seen that the resulting lower bound becomes

(27) $$\frac{E\{L\left[\phi_{n}\left(U^{n}\right)\right]\}}{n}\geq \frac{h(U^{n})}{n}-\Phi \left(D\right)-O\left(\frac{\log n}{n}\right),$$

which is in agreement with the subtraction of O((logn)/n) below the entropy for lossless one-to-one codes [11]. This reduction of O((logn)/n) is due to the fact that the class of one-to-one codes is broader than the class of UD codes, and therefore the former codes are potentially more capable for a given finite *n*, albeit the difference fades away as *n* grows.

### 4.2. D-Semifaithful Codes

For *D*-semifaithful codes with UD correspondence between the compressed bit-stream and the reproduction, we obtain from Lemma 3, combined with a derivation like (18),

(28) $$E\left\{L\left[\phi_{n}\left(U^{n}\right)\right]\right\}\geq h\left(U^{n}\right)-\log\mathrm{Vol}\left\{S_{n}\left(D\right)\right\},$$

which is in line with the results of [10], and the remaining issue becomes the assessment of the log-volume of $S_{n}\left(D\right)$ or an evaluation of a good upper bound to this quantity. One way to proceed is to apply a Chernoff bound, as was suggested by Campbell [9]. For U=R, this amounts to the following derivation:

(29) $$\begin{array}{ccc} \log\mathrm{Vol}\{S_{n}\left(D\right)\} & = & \log\mathrm{Vol}\{z:\;\rho (z)\leq nD\}\\ & = & \log\left[\int_{R^{n}}I\left\{\rho \left(z\right)\leq nD\right\}dz\right]\\ & \leq & \log\left(\underset{\beta \geq 0}{\inf}\int_{R^{n}}2^{\beta [nD-\rho (z)]}dz\right)\\ & = & n\cdot \underset{\beta \geq 0}{\inf}\left\{\beta D+\log\left(\int_{R}2^{-\beta \rho (z)}dz\right)\right\}\\ & = & n\Phi (D), \end{array}$$

and we are back to the ordinary SLB,

(30) $$\frac{E\{L\left[\phi_{n}\left(U^{n}\right)\right]\}}{n}\geq \frac{h(U^{n})}{n}-\Phi \left(D\right),$$

exactly as we had for UD lossless compression of the reproduction data, and once again, this derivation extends straightforwardly to the case of multiple simultaneous distortion constraints by considering β, *D*, and ρ(·) to be vectors rather than scalars, as mentioned before.

However, since the class of *D*-semifaithful codes is narrower (and hence more limited to a certain extent) than the class of codes that merely comply with an average distortion constraint, it is conceivable to expect a somewhat tighter (larger) lower bound. Indeed, this turns out to be the case if the log-volume of $S_{n}\left(D\right)$ is estimated using a more sophisticated analysis tool, namely, the saddle-point method (see, e.g., Chapter 5 in [14] and Chapter 3 in [15], as well as its extension to the multivariate case [16]).

To apply the saddle-point method, the idea is to represent the indicator function in the integrand of the second line in (29) as I{ρ(z)≤nD}=u(nD−ρ(z)), where u(t) is the unit step function, defined as

(31) $$u\left(t\right)=\left\{\begin{array}{cc} 0 & t<0\\ 1 & t\geq 0 \end{array}\right.$$

which in turn is represented as the inverse Laplace transform of the complex function $U\left(s\right)=\frac{1}{s}$, i.e.,

(32) $$u\left(t\right)=\frac{1}{2\pi i}\int_{Re\{s\}=c}\frac{e^{st}ds}{s},$$

where $i\overset{▵}{=}\sqrt{-1}$ and *c* is an arbitrary positive real. It follows that

(33) Vol{Sn(D)}=∫Rnu(nD−ρ(z))dz =∫Rndz2πi∫Re{s}=ces(nD−ρ(z))dss =12πi∫Re{s}=cesnDdss∫Rne−sρ(z)dz =12πi∫Re{s}=cesnDdss∫Rnexp−s∑t=1nρ(zt)dz =12πi∫Re{s}=cesnDdss∫Re−sρ(z)dzn =12πi∫Re{s}=cdssexpnsD+ln∫Re−sρ(z)dz.

This path integral in the complex plane complies with the general form $\int_{A}^{B}g\left(s\right)e^{nf(s)}ds$, which under certain regularity conditions can be approximated for large *n* (see, e.g., Equation (5.7.2) in [14]) according to

(34) $$\int_{A}^{B}g\left(s\right)e^{nf(s)}ds=e^{i\theta }\sqrt{\frac{2\pi }{n|f\prime \prime \left(s_{★}\right)|}}\cdot g\left(s_{★}\right)e^{nf(s_{★})}\cdot \left[1+O\left(\frac{1}{n}\right)\right],$$

provided that the functions *f* and *g* are independent of *n* and analytic within some connected region D that includes *A* and *B* (which are also independent of *n*). Here, $s_{★}\in D$ is a saddle-point, i.e., a point where $f\prime \left(s_{★}\right)=0$, $f\prime \prime \left(s_{★}\right)>0$ and $g(s_{★})\neq 0$. The angle θ is called the axis and is given by $\theta =(\pi -arg\left\{f\prime \prime \left(s_{★}\right)\right\})/2$. In our case, $f\left(s\right)=sD+\ln\left[\int_{R}e^{-s\rho (z)}dz\right]$, $g\left(s\right)=\frac{1}{s}$, $\theta =\frac{\pi }{2}$, and $s_{★}$ is the point at which $f^{\prime }$ vanishes, which is assumed strictly positive. But this point of zero derivative is also the point that minimizes the convex function *f* across the positive reals. It follows then that $\mathrm{Vol}\{S_{n}\left(D\right)\}$ can be approximated as follows:

(35) $$\mathrm{Vol}\left\{S_{n}\left(D\right)\right\}=\frac{1}{s_{★}\sqrt{2\pi n|f\prime \prime \left(s_{★}\right)|}}\cdot \exp\left\{n\left(s_{★}D+\ln\left[\int_{R}e^{-s_{★}\rho \left(z\right)}dz\right]\right)\right\}\cdot \left[1+O\left(\frac{1}{n}\right)\right].$$

As for the exponential factor, observe that

(36) $$\begin{array}{cc} & \exp\left\{n\left(s_{★}D+\ln\left[\int_{R}e^{-s_{★}\rho \left(z\right)}dz\right]\right)\right\}\\ = & \exp\left\{n\cdot \underset{s\geq 0}{\inf}\left(sD+\ln\left[\int_{R}e^{-s\rho (z)}dz\right]\right)\right\}\\ = & \exp_{2}\left\{n\left(\log_{2}e\right)\cdot \underset{s\geq 0}{\inf}\left(sD+\frac{1}{\log_{2}e}\cdot \log_{2}\left[\int_{R}2^{-s\rho \left(z\right)\log_{2}e}dz\right]\right)\right\}\\ = & \exp_{2}\left\{n\underset{s\geq 0}{\inf}\left(sD\log_{2}e+\log_{2}\left[\int_{R}2^{-s\rho \left(z\right)\log_{2}e}dz\right]\right)\right\}\\ = & \exp_{2}\left\{n\underset{\beta \geq 0}{\inf}\left(\beta D+\log_{2}\left[\int_{R}2^{-\beta \rho (z)}dz\right]\right)\right\}\\ = & 2^{n\Phi (D)}, \end{array}$$

and so,

(37) $$\mathrm{Vol}\left\{S_{n}\left(D\right)\right\}=\frac{2^{n\Phi (D)}}{s_{★}\sqrt{2\pi n|f\prime \prime \left(s_{★}\right)|}}\cdot \left[1+O\left(\frac{1}{n}\right)\right],$$

which yields

(38) $$\frac{\log\mathrm{Vol}\{S_{n}\left(D\right)\}}{n}=\Phi \left(D\right)-\frac{\log n}{2n}-O\left(\frac{1}{n}\right),$$

and then, following Equation (28), we end up with

(39) $$\frac{E\{L\left[\phi_{n}\left(U^{n}\right)\right]\}}{n}\geq \frac{h(U^{n})}{n}-\Phi \left(D\right)+\frac{\log n}{2n}+O\left(\frac{1}{n}\right).$$

We therefore observe that the SLB for *D*-semifaithful codes is given by the ordinary SLB plus redundancy, whose leading term is $\frac{\log n}{2n}$. This is in agreement with findings of [10], where the derivations corresponded to special cases for which simple geometric and/or combinatorial considerations facilitated the accurate characterization of $\log\mathrm{Vol}\{S_{n}\left(D\right)\}$, but here the conclusion is more general. Note that we could have been even more precise and also specified the $O\left(\frac{1}{n}\right)$ term to be $\frac{1}{n}\cdot \log\left[s_{★}\sqrt{2\pi |f\prime \prime \left(s_{★}\right)|}\right]+O\left(\frac{1}{n^{2}}\right)$, but this is, of course, less important.

When *k* simultaneous distortion constraints are imposed pointwise, i.e.,

(40) $$\max_{u^{n}\in R^{n}}\rho_{j}\left(u^{n}-\psi_{n}\left(\phi_{n}\left(u^{n}\right)\right)\right)\leq nD_{j},\;\;\;j=1,2,\ldots ,k,$$

then using the multivariate saddle-point method [16], the above derivation extends with the vector version of the definition of Φ(D) replacing the scalar one, and the pre-exponential factor of the saddle-point approximation becomes

$$\frac{{\left(2\pi /n\right)}^{k/2}}{S\sqrt{\det\{\mathrm{Hess}\left\{f\right\}{|}_{s_{★}}\}}},$$

where *S* is the product of the components of the *k*-dimensional vector $s_{★}$—the point at which ∇f(s)=0, where $f\left(s\right)=s\cdot D+\ln\left[\int_{R}e^{-s\cdot \rho (z)}dz\right]$ is defined such that *s*, *D*, and ρ(·) are *k*-dimensional vectors, with s·D and s·ρ(z) being inner products, as was defined before. Here, $\det\{\mathrm{Hess}\left\{f\right\}{|}_{s_{★}}$ is the determinant of the Hessian of *f*, computed at $s=s_{★}$. It is assumed, of course, that the k×k matrix ${\mathrm{Hess}\left\{f\right\}|}_{s_{★}}$ is non-singular; otherwise there might be redundant (inactive) constraints, which should be removed from the calculation. In this case, the redundancy on top of the ordinary SLB is $\frac{k^{\prime }\log n}{2n}+O\left(\frac{1}{n}\right)$, i.e.,

(41) $$\frac{E\{L\left[\phi_{n}\left(U^{n}\right]\right\}}{n}\geq \frac{h(U^{n})}{n}-\Phi \left(D\right)+\frac{k^{\prime }\log n}{2n}+O\left(\frac{1}{n}\right),$$

where $k^{\prime }\leq k$ is the effective dimension after the possible removal of redundant constraints.

For example, if k=2, $\rho_{1}\left(z\right)=\left|z\right|$, and $\rho_{2}\left(z\right)=z^{2}$, then obviously, ${\left(\frac{1}{n}\sum_{t=1}^{n}\left|z_{t}\right|\right)}^{2}$ cannot exceed $\frac{1}{n}\sum_{t=1}^{n}z_{t}^{2}$, and so, if $D_{1}>\sqrt{D_{2}}$, the constraint $\sum_{t=1}^{n}\left|z_{t}\right|\leq nD_{1}$ is inactive (and hence removable) in the presence of the constraint $\sum_{t=1}^{n}z_{t}^{2}\leq nD_{2}$. In this case, although k=2, the effective dimension is $k^{\prime }=1$. Another obvious example of a superfluous distortion constraint occurs when there is a linear dependence. For instance, let k=3 and $\rho_{3}\left(z\right)=a\rho_{1}\left(z\right)+b\rho_{2}\left(z\right)$, where *a* and *b* are fixed positive reals. Then, whenever $D_{3}\geq aD_{1}+bD_{2}$, the third distortion constraint is redundant and then $k^{\prime }=2$ (or even less, if there are additional superfluous constraints). More generally, inactive constraints can be identified as those whose corresponding components of $s_{★}$ vanish.

Another type of situation where the effective dimension $k^{\prime }$ is smaller than the formal dimension *k* occurs when the dependence of f(s) on some of the components of *s* disappears in the first place. Consider, for example, the case where k=2, $\rho_{1}\left(z\right)=z^{2}$, and the other distortion constraint is $\max_{1\leq t\leq n}\left|z_{t}\right|\leq A$; in other words, we impose both an average quadratic constraint and a peak-limited distortion constraint with the motivation of avoiding large spikes in the reconstruction error signal. The peak-limited distortion constraint can be represented as an additive distortion constraint if we select $\rho_{2}\left(z\right)=W\left(|z|-A\right)$, where W(·) is the IWF defined in (13) and the value of $D_{2}$ can then be selected to be an arbitrary finite, non-negative real, say $D_{2}=0$. Here again $k^{\prime }=1$, but as said, this time, it is because the function f(s) depends only on one component of the vector $s=(s_{1},s_{2})$ to begin with. To see this, observe that

(42) $$\begin{array}{ccc} f(s) & = & sD+\ln\left[\int_{R}e^{-s\rho (z)}dz\right]\\ & = & s_{1}D_{1}+s_{2}D_{2}+\ln\left[\int_{R}e^{-s_{1}\rho_{1}\left(z\right)+s_{2}\rho_{2}\left(z\right)}dz\right]\\ & = & s_{1}D_{1}+s_{2}\cdot 0+\ln\left[\int_{R}e^{-s_{1}z^{2}+s_{2}W\left(|z|-A\right)}dz\right]\\ & = & s_{1}D_{1}+\ln\left[\int_{-A}^{A}e^{-s_{1}z^{2}}dz\right]\\ & = & s_{1}D_{1}+\ln\left(\sqrt{\frac{\pi }{s_{1}}}\cdot \left[1-2Q\left(A\sqrt{2s_{1}}\right)\right]\right)\\ & = & s_{1}D_{1}+\frac{1}{2}\ln\left(\frac{\pi }{s_{1}}\right)+\ln\left[1-2Q\left(A\sqrt{2s_{1}}\right)\right], \end{array}$$

where Q(·) is the well-known *Q*-function, defined as

(43) $$Q\left(t\right)=\int_{t}^{\infty }\frac{e^{-x^{2}/2}dx}{\sqrt{2\pi }}.$$

Here, although formally there are k=2 distortion constraints, the saddle-point integration is over one complex variable only, and so the redundancy is $\frac{\log n}{2n}+O\left(\frac{1}{n}\right)$ on top of the ordinary SLB, $\frac{h(U^{n})}{n}+\Phi \left(D\right)$.

## 5. Sliding-Window Distortion Constraints

Consider a situation where one wishes not only to control the ‘intensity’ of the reconstruction error signal, $\sum_{t=1}^{n}\rho \left(z_{i}\right)$, but also possibly to shape its ‘continuity’ or its ‘smoothness’ by imposing additional limitations, say, on the empirical autocorrelations of the error signal, $z^{n}=\left(z_{1},z_{2},\ldots ,z_{n}\right)$, in order to suppress, for example, high frequencies, which might be disturbing for the human eye (in the case of images and video streams) or the human ear (in the case of audio signals). For instance, one might wish to constrain the error signal to obey the first lag autocorrelation constraint, $\frac{1}{n}\sum_{t=2}^{n}z_{t}z_{t-1}\geq 0.95$ (and perhaps also further lags), in addition to the ordinary mean-square error constraint, say, $\frac{1}{n}\sum_{t=1}^{n}z_{t}^{2}\leq 1$. More generally, consider the case where we impose *k* additive constraints pertaining to sliding-window functions of the form

(44) $$\sum_{t=m}^{n}\rho_{j}\left(z_{t-m+1}^{t}\right)\leq nD_{j},\;\;\;\;\;j=1,2,\ldots ,k,$$

where *m* is a positive integer that designates the size of the sliding window. For m=1, we are back to ordinary additive distortion constraints, where the single-letter distortion function $\rho_{j}$ operates on single symbols separately. If the sizes of the sliding window are different for the *k* various constraints, we take *m* to be the largest one. Accordingly, in the above example where k=2 and the constraints are $\frac{1}{n}\sum_{t=1}^{n}z_{t}^{2}\leq 1$ and $\frac{1}{n}\sum_{t=2}^{n}z_{t}z_{t-1}\geq 0.95$, we have $\rho_{1}\left(z\right)=z^{2}$, $D_{1}=1$, $\rho_{2}\left(z_{1},z_{2}\right)=-z_{1}\cdot z_{2}$, and $D_{2}=-0.95$. In this case, the sliding-window size is m=2, and the minus signs in the correlation constraint are due to the reversal of the direction of the inequality in $\frac{1}{n}\sum_{t=2}^{n}z_{t}z_{t-1}\geq 0.95$, as opposed to the direction of inequalities in our distortion constraints in general.

How does the SLB extend to the case of sliding-window constraints of this type? In this section, we assume that *m* is fixed while *n* tends to infinity, and we focus merely on the main terms of the resulting SLB, disregarding the redundancy terms.

A straightforward extension of Lemma 1 and the subsequent derivation in Section 4, so as to apply to a set of *k* sliding-window distortion constraints with window size *m*, yields the lower bound

(45) $$E\left\{L\left[\phi \left(U^{n}\right)\right]\right\}\geq h\left(U^{n}\right)-\underset{\beta \in {\left[0,\infty \right)}^{k}}{\inf}\left(\log\left[\int_{U^{n}}\exp_{2}\left\{-\beta \cdot \sum_{t=m}^{n}\rho \left(z_{t-m+1}^{t}\right)\right\}dz^{n}\right]+n\beta \cdot D\right),$$

where $\beta =(\beta_{1},\ldots ,\beta_{k})$, $D=(D_{1},\ldots ,D_{k})$, $\rho \left(z_{t-m+1}^{t}\right)=\left(\rho_{1}\left(z_{t-m+1}^{t}\right),\ldots ,\rho_{k}\left(z_{t-m+1}^{t}\right)\right)$, and the dot operations are understood as inner products, as defined earlier.

The multi-dimensional integral

(46) $$\int_{U^{n}}\exp_{2}\left\{-\beta \cdot \sum_{t=m}^{n}\rho \left(z_{t-m+1}^{t}\right)\right\}dz^{n}=\int_{U^{n}}\prod_{t=m}^{n}\exp_{2}\left\{-\beta \cdot \rho (z_{t-m+1}^{t})\right\}dz^{n}$$

can be viewed as being obtained by iterated applications of a sliding-window integral operator whose kernel is given by $K_{\beta }\left(z_{t-m+1}^{t}\right)=\exp_{2}\left\{-\beta \cdot \rho (z_{t-m+1}^{t})\right\}$ and the integration is over one component of $z^{n}$ at a time. Under certain regularity conditions, the value of this multi-dimensional integral grows exponentially with an exponential order of ${\left[\lambda \left(\beta \right)\right]}^{n}$, where λ(β) is the spectral radius of the operator kernel, namely, the dominant eigenvalue, and then the asymptotic form of the SLB becomes

(47) $$\underset{n\to \infty }{lim inf}\frac{E\{L\left[\phi_{n}\left(U^{n}\right)\right]\}}{n}\geq \bar{h}\left(U^{\infty }\right)-\underset{\beta \in {\left[0,\infty \right)}^{k}}{\inf}\left[\log\lambda \left(\beta \right)+\beta D\right].$$

There are several equivalent formulas for calculating the spectral radius, λ(β), of a sliding-window kernel $K_{\beta }\left(\cdot \right)$ for a given β, for example, the Collatz–Wielandt formula [17,18] and the Donsker–Varadhan formula [19]. Both formulas are associated with calculus of variations. For details, see, e.g., Subsection 3.4 of [20]. In certain special cases, such as those that involve a symmetric kernel for m=2, $K_{\beta }\left(z,z^{\prime }\right)$, the Rayleigh quotient formula

(48) $$\lambda \left(\beta \right)=\underset{g}{\sup}\frac{\int_{U^{2}}g\left(z\right)K_{\beta }\left(z,z^{\prime }\right)g\left(z^{\prime }\right)dzdz^{\prime }}{\int_{U}g^{2}\left(z\right)dz}.$$

can also be used. Note that even in this relatively simple special case, the evaluation of λ(β) is a problem of calculus of variations and hence it is not trivial computationally. Clearly, in the finite-alphabet case, the operator kernel reduces to a finite-dimensional matrix, and then the spectral radius is simply the Perron–Frobenius eigenvalue (see, e.g., Theorem 8.2.11 of [21]).

As a simple example, consider the case k=m=2, $\rho_{1}\left(z\right)=z^{2}$, $D_{1}=D$, $\rho_{2}\left(z,z^{\prime }\right)=-z\cdot z^{\prime }$, and $D_{2}=-\theta$, where D>0 and θ∈[0,1) are given parameters. Then, following the analogous derivation of Example 4 in [20], we find that the resulting SLB is given by

(49) $$\underset{n\to \infty }{lim inf}\frac{E\{L\left[\phi_{n}\left(U^{n}\right)\right]\}}{n}\geq \bar{h}\left(U^{\infty }\right)-\frac{1}{2}\log\left[2\pi eD\left(1-\theta^{2}\right)\right],$$

or, in words, the rate penalty due to the additional autocorrelation constraint is $\frac{1}{2}\log\frac{1}{1-\theta^{2}}$ on top of the SLB pertaining to the ordinary quadratic distortion constraint, $\frac{h(U^{n})}{n}-\frac{1}{2}\log\left(2\pi eD\right)$.

## 6. Individual Sequences and Finite-State Encoders

We conclude the technical part of this article by deriving an individual-sequence counterpart of the SLB for finite-alphabet, deterministic sequences, which is based on a variation of Ziv and Lempel’s generalized Kraft inequality (see Lemma 2 of [13]).

Generally speaking, our model for lossy compression of individual sequences is based on the following simple structure. Each source block of length *m*, $u^{m}\in U^{m}$ (*m*—positive integer) is first mapped by an arbitrary reproduction encoder (or vector quantizer) into a reproduction vector,

(50) $$v^{m}=q\left(u^{m}\right)\in V_{m}\subseteq U^{m}$$

and then the concatenation of the resulting *m*-vectors, $\left\{v^{m}\right\}$, forming the sequence $v_{1},v_{2},\ldots$, is compressed losslessly by a finite-state encoder following the model of [13]. Note that in general, the adoption of this structure does not harm generality and optimality because the mapping between the reproduction vectors and the compressed bit-streams should be bijective (as otherwise additional distortion is injected in the compression phase), and so one may envision a scenario where the encoder holds a copy of the reproduction codebook and cascades it with lossless compression of the reproduction vectors.

To keep this article self-contained, we begin with some basic background on lossless compression of individual sequences by finite-state encoders and the 1978 Lempel–Ziv (LZ78) algorithm. Readers familiar with this background may safely skip Section 6.1 and move on directly to Section 6.2.

### 6.1. Background

Following the model of [13], consider a setting of lossless compression of $v^{n}$ on the basis of finite-state (FS) encoders. An FS encoder is defined by the set E=(U,Y,Σ,f,g), where U is the finite alphabet of each symbol, $v_{i}$, which is the same as the source alphabet and whose size is *r*; Y is a finite collection of binary, variable-length strings, which is allowed to consist of an empty string λ (whose length is zero); Σ is a set of *s* states of the encoder; f:Σ×U→Y is the output function; and g:Σ×U→Σ is the next-state function. Given an infinite input reproduction vector (obtained by concatenating infinitely many output vectors from the reproduction encoder), $v=(v_{1},v_{2},\ldots )$ with $v_{i}\in U$, i=1,2,…, the FS encoder *E* produces an infinite output sequence, $y=(y_{1},y_{2},\ldots )$ with $y_{i}\in Y$, henceforth referred to as the compressed bit-stream, while passing through a sequence of states $\sigma =(\sigma_{1},\sigma_{2},\ldots )$ with $\sigma_{i}\in \Sigma$. The encoder is governed recursively by the equations

(51) $$\begin{array}{ccc} y_{i} & = & f(\sigma_{i},v_{i}), \end{array}$$

(52) $$\begin{array}{ccc} \sigma_{i+1} & = & g(\sigma_{i},v_{i}), \end{array}$$

for i=1,2,…, with a fixed initial state $\sigma_{1}=\sigma_{★}\in Z$. If at any step $y_{i}=\lambda$, this is referred to as idling, as no output is generated, but only the state is kept updated in response to the input.

An encoder with *s* states, henceforth called an *s*-state encoder, is one for which |Σ|=s. For the sake of simplicity, we adopt a few notation conventions from [13]: Given a segment of input symbols $v_{i}^{j}$ with i≤j and an initial state $\sigma_{i}$, we use $f(\sigma_{i},v_{i}^{j})$ to denote the corresponding output segment $y_{i}^{j}$ produced by *E*. Similarly, $g(\sigma_{i},v_{i}^{j})$ will denote the final state $\sigma_{j+1}$ after processing the inputs $v_{i}^{j}$, beginning from state $\sigma_{i}$.

An FS encoder *E* is called information lossless (IL) if, given any initial state $\sigma_{i}\in \Sigma$, any positive integer *n*, and any input string, $v_{i}^{i+n}$, the set $\left(\sigma_{i},f\left(\sigma_{i},v_{i}^{i+n}\right),g\left(\sigma_{i},v_{i}^{i+n}\right)\right)$ uniquely determines the corresponding input string $v_{i}^{i+n}$.

The incremental parsing process used by the LZ78 algorithm is a sequential procedure for processing a finite-alphabet input $u^{n}$. At each step of this process, one determines the shortest string that has not yet occurred as a complete phrase in the current parsed set, with the possible exception of the last phrase, which might be incomplete. For example, applying this parsing method to the sequence

$$u^{15}=011010011000100$$

yields

0,1,10,100,11,00,01,00.

Let us denote by $c(u^{n})$ the total number of distinct phrases generated by this procedure (here, $c(u^{15})=8$). In addition, let $LZ(u^{n})$ represent the length in bits of the binary string produced by the LZ78 encoding of $u^{n}$. By Theorem 2 in [13], the following inequality holds:

(53) $$LZ\left(u^{n}\right)\leq \left[c\left(u^{n}\right)+1\right]\log\left\{2r\left[c\left(u^{n}\right)+1\right]\right\}$$

which can easily be shown to be further upper bounded by

(54) $$LZ\left(u^{n}\right)\leq c\left(u^{n}\right)\log c\left(u^{n}\right)+n\cdot \epsilon_{1}\left(n\right),$$

where $\epsilon_{1}\left(n\right)$ tends to zero uniformly as n→∞. In words, the LZ78 code length for $v^{n}$ is upper bounded by an expression whose leading term is $c\left(u^{n}\right)\log c\left(u^{n}\right)$.

We shall refer to the quantity $c\left(u^{n}\right)\log c\left(u^{n}\right)$ as the unnormalized LZ complexity of $u^{n}$ to distinguish from the normalized LZ complexity defined as $\frac{c\left(u^{n}\right)\log c\left(u^{n}\right)}{n}$, which means the per-symbol LZ complexity.

### 6.2. SLB for Individual Sequences

The following lemma provides a variation of the generalized Kraft inequality of [13].

**Lemma** **5.** *For every IL FS encoder with s states, every α>1, every β≥0, and every positive integer ℓ, which is an integer multiple of m,*

(55) $$\sum_{\sigma \in \Sigma }\sum_{w^{l}\in U^{l}}\exp_{2}\left\{-\alpha L\left[f\left(\sigma ,q\left(w^{l}\right)\right)\right]-\beta \rho \left(w^{l}-q\left(w^{l}\right)\right)\right\}\leq \frac{s^{2}{\left[\sum_{z\in U}2^{-\beta \rho (z)}\right]}^{l}}{2^{\alpha -1}-1},$$

*where* $q\left(w^{l}\right)\equiv q\left(w_{1}^{m},w_{m+1}^{2m},\ldots ,w_{l-m+1}^{l}\right)\overset{▵}{=}\left(q\left(w_{1}^{m}\right),q\left(w_{m+1}^{2m}\right),\ldots ,q\left(w_{l-m+1}^{l}\right)\right)$*, the latter being defined as in (50) for vectors in* $U^{l}$*.*

**Proof.**  From the postulated IL property, it follows that given σ∈Σ, there cannot be more than $s2^{k}$ distinct vectors, $\left\{v^{l}\right\}$, such that $L[f\left(\sigma ,v^{l}\right)]=k$ for every positive integer *k*. Therefore,

(56) $$\begin{array}{cc} & \sum_{\sigma \in \Sigma }\sum_{w^{l}\in U^{l}}\exp_{2}\left\{-\alpha L\left[f\left(\sigma ,q\left(w^{l}\right)\right)\right]-\beta \rho \left(w^{l}-q\left(w^{l}\right)\right)\right\}\\ = & \sum_{\sigma \in \Sigma }\sum_{k\geq 1}\sum_{\left\{v^{l}:\;L\left[f\left(\sigma ,v^{l}\right)\right]=k\right\}}\sum_{\left\{w^{l}:\;q\left(w^{l}\right)=v^{l}\right\}}\exp_{2}\left\{-\alpha k-\beta \rho (w^{l}-q\left(w^{l}\right))\right\}\\ \leq & \sum_{\sigma \in \Sigma }\sum_{k\geq 1}s\cdot 2^{k}\cdot 2^{-\alpha k}\sum_{z^{l}\in U^{l}}2^{-\beta \rho (z^{l}))}\\ = & s^{2}\cdot {\left[\sum_{u\in U}2^{-\beta \rho (z)}\right]}^{l}\cdot \sum_{k=1}^{\infty }2^{-(\alpha -1)k}\\ = & \frac{s^{2}{\left[\sum_{z\in U}2^{-\beta \rho (z)}\right]}^{l}}{2^{\alpha -1}-1}. \end{array}$$

This completes the proof of Lemma 5. □

Now, let *ℓ* divide *n*. For a given FS IL encoder *E*, a given $u^{n}\in U^{n}$, and the associated state sequence $\sigma^{n}\in \Sigma^{n}$ (generated from $u^{n}$ by *E* using the next-state function *g* recursively), consider the joint empirical distribution

(57) $$\hat{P}\left(\sigma ,w^{l}\right)=\frac{l}{n}\sum_{i=0}^{n/l-1}I\left\{\sigma_{il+1}=\sigma ,\;u_{il+1}^{il+l}=w^{l}\right\},\;\;\;\;\sigma \in \Sigma ,\;w^{l}\in U^{l}$$

Then, according to Lemma 5,

(58) $$\begin{array}{cc} & \frac{s^{2}{\left[\sum_{z\in U}2^{-\beta \rho (z)}\right]}^{l}}{2^{\alpha -1}-1}\\ \geq & \sum_{\sigma \in \Sigma }\sum_{w^{l}\in U^{l}}\exp_{2}\left\{-\alpha L\left[f\left(\sigma ,q\left(w^{l}\right)\right)\right]-\beta \rho \left(w^{l}-q\left(w^{l}\right)\right)\right\}\\ = & \sum_{\sigma \in \Sigma }\sum_{w^{l}\in U^{l}}\hat{P}\left(\sigma ,w^{l}\right)\exp_{2}\left\{-\alpha L\left[f\left(\sigma ,q\left(w^{l}\right)\right)\right]-\beta \rho \left(w^{l}-q\left(w^{l}\right)\right)-\log\hat{P}\left(\sigma ,w^{l}\right)\right\}\\ \geq & \exp_{2}\left\{-\alpha \sum_{\sigma \in \Sigma }\sum_{w^{l}\in U^{l}}\hat{P}\left(\sigma ,w^{l}\right)L\left[f\left(\sigma ,q\left(w^{l}\right)\right)\right]-\beta \sum_{w^{l}\in U^{l}}\hat{P}\left(w^{l}\right)\rho \left(w^{l}-q\left(w^{l}\right)\right)+\hat{H}\left(S,U^{l}\right)\right\}\\ = & \exp_{2}\left\{-\alpha \cdot \frac{l}{n}\sum_{i=0}^{n/l-1}L\left[f\left(\sigma_{il+1},v_{il+1}^{il+l}\right)\right]-\beta \cdot \frac{l}{n}\sum_{i=0}^{n/l-1}\rho \left(u_{il+1}^{il+l}-q\left(u_{il+1}^{il+l}\right)\right)+\hat{H}\left(S,U^{l}\right)\right\}\\ = & \exp_{2}\left\{-\alpha \cdot \frac{l}{n}\sum_{t=1}^{n}L\left[f\left(\sigma_{t},v_{t}\right)\right]-\beta \cdot \frac{l}{n}\sum_{t=1}^{n}\rho \left(u_{t}-v_{t}\right)+\hat{H}\left(S,U^{l}\right)\right\}, \end{array}$$

where $\hat{P}\left(w^{l}\right)=\sum_{\sigma \in \Sigma }\hat{P}\left(\sigma ,w^{l}\right)$ and $\hat{H}\left(S,U^{l}\right)$ is the joint entropy of the auxiliary random variables S∈Σ and $U^{l}\in U^{l}$, induced by the empirical joint distribution $\hat{P}$. It follows then that

(59) $$\begin{array}{ccc} & & \alpha \cdot \frac{1}{n}\sum_{t=1}^{n}L\left[f\left(\sigma_{t},v_{t}\right)\right]+\beta \cdot \frac{1}{n}\sum_{t=1}^{n}\rho \left(u_{t}-v_{t}\right)\\ & \geq & \frac{\hat{H}\left(S,U^{l}\right)}{l}-\frac{\log(s^{2})}{l}-\log\left[\sum_{z\in U}2^{-\beta \rho (z)}\right]+\frac{\log(2^{\alpha -1}-1)}{l}\\ & \geq & \frac{\hat{H}\left(U^{l}\right)}{l}-\log\left[\sum_{z\in U}2^{-\beta \rho (z)}\right]-\frac{2\log s}{l}+\frac{\log(2^{\alpha -1}-1)}{l}\\ & \geq & \frac{c\left(u^{n}\right)\log c\left(u^{n}\right)}{n}-\log\left[\sum_{z\in U}2^{-\beta \rho (z)}\right]-\Delta_{n}\left(l\right)-\frac{2\log s}{l}+\frac{\log(2^{\alpha -1}-1)}{l}, \end{array}$$

where $\lim_{n\to \infty }\Delta_{n}\left(l\right)=\frac{1}{l}$, and the last step can be found in Equation (12) of [22] as well as references therein. By taking the limit of l→∞ (followed by the limit n→∞), the last three terms can be made arbitrarily small, whereas the first two terms serve as the individual-sequence counterpart of the right-hand side of Equation (20). Moving the distortion term to the right-hand side and optimizing over β, we obtain

(60) $$\begin{array}{c} \alpha \cdot \frac{1}{n}\sum_{t=1}^{n}L\left[f\left(\sigma_{t},v_{t}\right)\right]\geq \frac{c\left(u^{n}\right)\log c\left(u^{n}\right)}{n}-\underset{\beta \geq 0}{\inf}\left\{\beta \cdot \frac{1}{n}\sum_{t=1}^{n}\rho \left(u_{t}-v_{t}\right)+\log\left[\sum_{z\in U}2^{-\beta \rho (z)}\right]\right\}-\\ \Delta_{n}\left(l\right)-\frac{2\log s}{l}+\frac{\log(2^{\alpha -1}-1)}{l}\\ =\frac{c\left(u^{n}\right)\log c\left(u^{n}\right)}{n}-\Phi \left(\frac{1}{n}\sum_{t=1}^{n}\rho \left(u_{t}-v_{t}\right)\right)-\\ \Delta_{n}\left(l\right)-\frac{2\log s}{l}+\frac{\log(2^{\alpha -1}-1)}{l}. \end{array}$$

Let $L_{\max}=\max_{\sigma ,v}L\left[f\left(\sigma ,v\right)\right]$. Selecting $\alpha =1+\zeta_{l}$, where $\zeta_{l}$ tends to zero at a sub-exponential rate (say, $\zeta_{l}=1/l$), we have, on the one hand, the previous inequality and, on the other hand,

(61) $$\alpha \cdot \frac{1}{n}\sum_{t=1}^{n}L\left[f\left(\sigma_{t},v_{t}\right)\right]\leq \frac{1}{n}\sum_{t=1}^{n}L\left[f\left(\sigma_{t},v_{t}\right)\right]+\zeta_{l}\cdot L_{\max},$$

and so our main result in this section is the following:

(62) $$\begin{array}{ccc} \frac{1}{n}\sum_{t=1}^{n}L\left[f\left(\sigma_{t},v_{t}\right)\right] & \geq & \frac{c\left(u^{n}\right)\log c\left(u^{n}\right)}{n}-\Phi \left(\frac{1}{n}\sum_{t=1}^{n}\rho \left(u_{t}-v_{t}\right)\right)-\\ & & \Delta_{n}\left(l\right)-\frac{2\log s}{l}+\frac{\log(2^{\zeta_{l}}-1)}{l}-\zeta_{l}\cdot L_{\max}\\ & \geq & \frac{c\left(u^{n}\right)\log c\left(u^{n}\right)}{n}-\Phi \left(\frac{1}{n}\sum_{t=1}^{n}\rho \left(u_{t}-v_{t}\right)\right)-\\ & & \Delta_{n}\left(l\right)-\frac{2\log s}{l}+\frac{\log(\zeta_{l}\ln2)}{l}-\zeta_{l}\cdot L_{\max}, \end{array}$$

with the last four terms tending to zero in the limit of l→∞ followed by the limit n→∞.

To conclude, the individual-sequence counterpart of the SLB for finite-alphabet source sequences is essentially of the same form as the classical SLB of the probabilistic setting, except that the normalized entropy term is replaced by the normalized LZ complexity of the sequence and the function Φ(·) is calculated at the point of the actual distortion, $\frac{1}{n}\sum_{t=1}^{n}\rho \left(u_{t}-v_{t}\right)$.

## 7. Summary and Conclusions

In this article, we have derived several modified versions of the Kraft inequality for lossy compression, which allowed us to derive a few sharper forms and broader types of the classical SLB. This was performed in a unified framework that cover various classes of rate-distortion coding schemes and types of distortion constraints. Two classes of codes were considered: one-to-one codes and *D*-semifaithful codes (as well as codes that are both one-to-one and *D*-semifaithful). The other types of distortion constraints are sliding-window constraints. Finally, we also derived an individual-sequence counterpart of the Shannon lower bound, which relies on an extended Kraft inequality for IL FS encoders.

It is pointed out that some of the above-mentioned refinements and extensions required us to invoke more advanced analysis tools than those transitionally used in Shannon theory, such as the saddle-point method (in the context of *D*-semifaithful codes) and the spectral theory of linear operators (in the context of sliding-window distortion constraints).

## Data Availability

No new data were created or analyzed in this study.

## Appendix A

**Proof of the Second Equality in Equation** **(4).** Let us define the density function,

(A1) $$g\left(z\right)=\frac{2^{-\beta \rho (z)}}{\int_{U}2^{-\beta \rho (z^{\prime })}dz\prime }.$$

Then,

(A2) $$\begin{array}{ccc} \underset{\beta \geq 0}{\inf}\left\{\log\left[\int_{U}2^{-\beta \rho (z)}dz\right]+\beta D\right\} & = & \underset{\beta \geq 0}{\inf}\left\{\underset{f}{\sup}\left[-D\left(f∥g\right)\right]+\log\left[\int_{U}2^{-\beta \rho (z)}dz\right]+\beta D\right\}\\ & = & \underset{\beta \geq 0}{\inf}\left\{\underset{f}{\sup}\left[\int_{U}dzf\left(z\right)\log\left(\frac{2^{-\beta \rho (z)}}{f(z)}\right)\right]+\beta D\right\}\\ & = & \underset{\beta \geq 0}{\inf}\underset{f}{\sup}\left\{-\int_{U}dzf\left(z\right)\log f\left(z\right)+\beta \left[D-\int_{U}f\left(z\right)\rho \left(z\right)dz\right]\right\}\\ & \overset{\left(a\right)}{=} & \underset{f}{\sup}\underset{\beta \geq 0}{\inf}\left\{-\int_{U}dzf\left(z\right)\log f\left(z\right)+\beta \left[D-\int_{U}f\left(z\right)\rho \left(z\right)dz\right]\right\}\\ & = & \underset{f}{\sup}\underset{\beta \geq 0}{\inf}\left\{h(Z)+\beta [D-E\{\rho (Z)\}]\right\}\\ & = & \underset{f}{\sup}\left\{\begin{array}{cc} -\infty & E\{\rho (Z)\}>D\\ h(Z) & E\{\rho (Z)\}\leq D \end{array}\right.\\ & = & \underset{\left\{Z:\;E\{\rho (Z)\}\leq D\right\}}{\sup}h\left(Z\right), \end{array}$$

where (a) is due to the fact that the objective function, $-\int_{U}dzf\left(z\right)\log f\left(z\right)+\beta \left[D-\int_{U}f\left(z\right)\rho \left(z\right)dz\right]$, is concave in *f* and affine (and hence convex) in β. □

## References

[1] Berger T. (1971). Rate Distortion Theory—A Mathematical Basis for Data Compression.

[2] Gray R.M. (1990). Source Coding Theory.

[3] Covet T.M., Thomas J.A. (2006). Elements of Information Theory.

[4] Linder T., Zamir R. (1994). On the asymptotic tightness of the Shannon lower bound. IEEE Trans. Inf. Theory.

[5] Koch T. (2016). The Shannon lower bound is asymptotically tight. IEEE Trans. Inf. Theory.

[6] Kostina V. Data compression with low distortion and finite blocklength. Proceedings of the 2015 53rd Annual Allerton Conference on Communication, Control, and Computing (Allerton).

[7] Kostina V. When is Shannon’s lower bound tight at finite blocklength?. Proceedings of the 2016 54th Annual Allerton Conference on Communication, Control, and Computing (Allerton).

[8] Gastpar M., Sula E. (2024). Shannon bounds for quadratic rate-distortion problems. IEEE J. Sel. Areas Inf. Theory.

[9] Campbell L.L. (1973). Kraft inequality for decoding with respect to a fidelity criterion. IEEE Trans. Inf. Theory.

[10] Merhav N. (1995). A comment on “A rate of convergence result for a universal *D*-semifaithful code”. IEEE Trans. Inf. Theory.

[11] Rissanen J. (1982). Tight lower bounds for optimum code length. IEEE Trans. Inf. Theory.

[12] Zhang Z., Yang E.-h., Wei V.K. (1997). The redundancy of source coding with a fidelity criterion. I. known statistics. IEEE Trans. Inf. Theory.

[13] Ziv J., Lempel A. (1978). Compression of individual sequences via variable-rate coding. IEEE Trans. Inf. Theory.

[14] de Bruijn N.G. (1981). Asymptotic Methods in Analysis.

[15] Merhav N., Weinberger N. (2025). A toolbox for refined information-theoretic analyses with applications. Found. Trends Commun. Inf. Theory.

[16] Neuschel T. (2014). Apéry polynomials and the multivariate saddle point method. Contr. Approx..

[17] Collatz L. (1942). Einschlieβungssatz für die charakteristischen Zahlen von Matrizen. Math. Z..

[18] Wielandt H. (1950). Unzerlegbare, nicht negative Matrizen. Math. Z..

[19] Donsker M.D., Varadhan S.R.S. (1983). Asymptotic evaluation of certain Markov process expectations for large time, IV. Commun. Pure Appl. Math..

[20] Merhav N., Shamai S. (2025). Volume-based lower bounds to the capacity of the Gaussian channel under pointwise additive input constraints. arXiv.

[21] Horn R.A., Johnson C.R. (1985). Matrix Analysis.

[22] Merhav N. (2025). Universal Slepian-Wolf coding for individual sequences. IEEE Trans. Inf. Theory.

